# Effect of 5% lidocaine-medicated plaster in preventing chronic postsurgical pain after knee replacement: protocol for a randomized double-blind placebo-controlled trial

**DOI:** 10.3389/fphar.2024.1486217

**Published:** 2024-10-24

**Authors:** Junpeng Yuan, Huichan Xu, Jiongjiong Guo, Yan Li, Youjia Yu, Xiaohong Jin

**Affiliations:** ^1^ Department of Pain Medicine, The First Affiliated Hospital of Soochow University, Suzhou, China; ^2^ Department of Pain Medicine, Suzhou Xiangcheng People’s Hospital, Suzhou, China

**Keywords:** chronic postsurgical pain, knee arthroplasty, 5% lidocaine-medicated plaster, randomized controlled trial, multimodal analgesia, WOMAC index

## Abstract

**Background:**

With an aging population, knee arthroplasty is increasingly common; however, chronic postsurgical pain (CPSP) affects up to 30% of patients. This study aims to evaluate the efficacy of 5% lidocaine-medicated plaster (LP5) in preventing CPSP among patients undergoing knee arthroplasty.

**Methods:**

This is a dual-center, randomized, double-blind, placebo-controlled trial involving 128 adult patients scheduled for knee arthroplasty. Participants will be randomly assigned, stratified by center, to either the LP5 group or the placebo group (n = 64 per group). The LP5 or placebo group will apply the patch 1 day before surgery and on postoperative days 1–3, with multimodal analgesia administered postoperatively. Multimodal analgesia will include intraoperative flurbiprofen axetil and postoperative patient-controlled sufentanil. The primary outcome is the pain subscale of the Western Ontario and McMaster Universities Osteoarthritis Index (WOMAC) at 3 months postoperatively. Secondary outcomes will include WOMAC stiffness, function, and total scales; Leeds Assessment of Neuropathic Symptoms and Signs (LANSS) Pain Scale; 36-Item Short Form Health Survey (SF-36); postoperative pain scores; Visual Analog Scale (VAS) sleep scores; postoperative sufentanil consumption; need for rescue analgesia; length of Post-Anesthesia Care Unit (PACU) stay; length of hospital stay; and 90-day mortality. Safety outcomes will include assessments of hypotension, hypertension, bradycardia, tachycardia, arrhythmia, interventions for haemodynamic events, headache, dizziness, nausea, vomiting, local skin allergy, wound infection, and toxic reaction. Data will be analyzed following a modified intention-to-treat approach.

**Discussion:**

This study aims to provide high-quality evidence for the efficacy and safety of LP5 in preventing CPSP in patients undergoing knee arthroplasty.

## 1 Introduction

As the population ages, the prevalence of knee osteoarthritis continues to rise, resulting in an increasing number of knee arthroplasty procedures (Nham et al., 2023). Although knee arthroplasty is a definitive treatment for this condition and offers significant benefits, chronic postsurgical pain (CPSP) remains a significant issue, affecting 10%–30% of patients undergoing the procedure (Beswick et al., 2012). CPSP not only diminishes the quality of life and surgical outcomes for patients but also escalates healthcare costs (Schug et al., 2019). Treating CPSP faces high failure rates, with systemic medications such as antidepressants and antiepileptics providing only limited efficacy and being associated with significant adverse effects (Steyaert and Lavand’homme, 2018).

The 5% lidocaine-medicated plaster (LP5) is a topical analgesic that provides pain relief and local anesthesia by inhibiting voltage-gated sodium channels in neurons (Baron et al., 2016). It has shown significant efficacy in treating postherpetic neuralgia and diabetic peripheral neuropathic pain (Pei-Lin et al., 2008; White et al., 2010). Research indicates that lidocaine in LP5 can penetrate the stratum corneum to reach peripheral nerve fiber endings, inhibiting the transmission of persistent nociceptive signals from peripheral nerves and reducing the sensitization and hyperexcitability of neurons in both the peripheral and central nervous systems (Maloney et al., 2021). Existing studies have shown that LP5 is an effective treatment for chronic localized neuropathic pain following knee surgery. Beginning on day 7 of use, 83% of patients (20/24) experienced a 50% reduction in dynamic mechanical allodynia within 3 months (Pickering et al., 2019). However, the effectiveness of LP5 in preventing CPSP following knee arthroplasty remains unclear. Most current research focuses on treating chronic pain, whereas our study emphasizes early intervention to prevent pain, particularly given the refractory nature of CPSP. This study aims to evaluate the efficacy of LP5 in preventing CPSP in patients undergoing knee arthroplasty. By thoroughly examining the impact of LP5 on CPSP, we aim to provide valuable insights and potentially establish a new standard for postoperative pain management, thereby improving patient outcomes.

## 2 Methods

This protocol adheres to the Standard Protocol Items: Recommendations for Interventional Trials (SPIRIT) guidelines, as detailed in Supplementary Material.

### 2.1 Study design and patients

**FIGURE 1:**
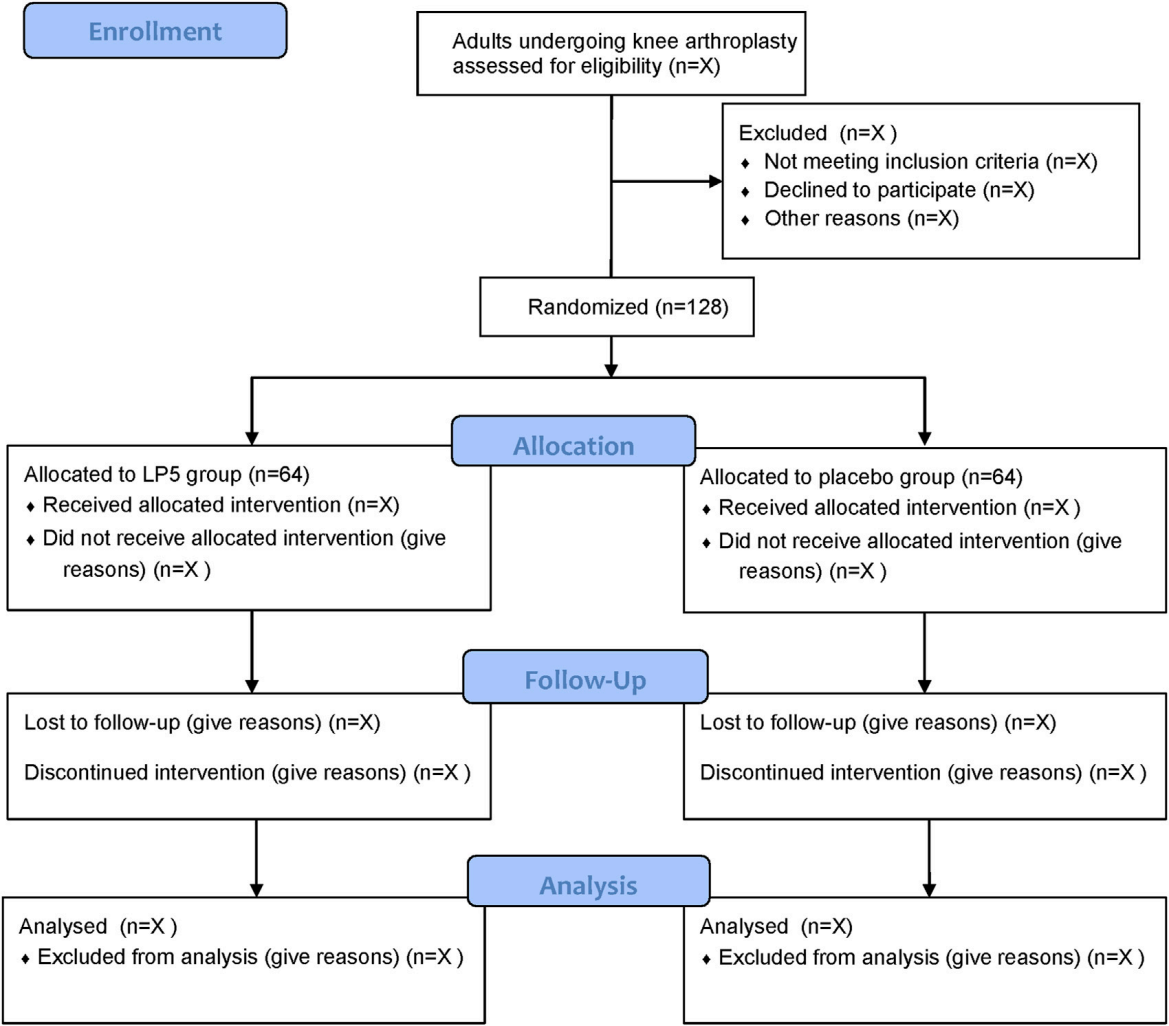
Study flow diagram. LP5, 5% lidocaine-medicated plaster.

This study is a dual-center, prospective, randomized, double-blind, placebo-controlled, parallel-group clinical trial. The trial will be conducted at the First Affiliated Hospital of Soochow University and Suzhou Xiangcheng People’s Hospital, enrolling a total of 128 patients. Recruitment is scheduled to take place from 16 July 2024 to 16 January 2025. The study flow diagram is shown in Figure 1.

### 2.2 Inclusion criteria

Patients who meet the following criteria will be included.

• Age ≥60 years;
• American Society of Anesthesiologists (ASA) physical status I-III;
• Body mass index (BMI) <35 kg/m^2^;
• Scheduled for unilateral first knee replacement surgery;
• Informed consent provided by patients and their families.

### 2.3 Exclusion criteria

The exclusion criteria include.

• A known allergy to lidocaine or adhesives;
• The presence of other severe chronic pain conditions;
• Long-term opioid use;
• A history of alcoholism;
• Severe liver or kidney insufficiency;
• A history of neurological or mental illness;
• Severe visual, hearing, or speech impairments that prevent the completion of assessment.

### 2.4 Primary outcome

The primary outcome will be the Western Ontario and McMaster Universities Osteoarthritis Index (WOMAC) Pain Scale, assessed via telephone or in-person interviews at 3 months postoperatively. The WOMAC Pain score evaluates pain severity across five activities: walking, using stairs, sitting or lying down, standing upright, and being in bed. Responses range from “none” to “extreme,” which are then summed and transformed into a 0–50 scale (Bellamy et al., 1988).

### 2.5 Secondary outcomes

Secondary outcomes will include WOMAC stiffness, function, and total scales; Leeds Assessment of Neuropathic Symptoms and Signs (LANSS) Pain Scale; 36-Item Short Form Health Survey (SF-36); postoperative pain scores; Visual Analog Scale (VAS) sleep scores; postoperative sufentanil consumption; need for rescue analgesia; length of Post-Anesthesia Care Unit (PACU) stay; length of hospital stay; and 90-day mortality.

### 2.6 Safety outcomes

Safety outcomes will assess hypotension, hypertension, bradycardia, tachycardia, arrhythmia, interventions for hemodynamic events, headache, dizziness, nausea, vomiting, local skin allergy, wound infection, and toxic reactions.

### 2.7 Randomization and blinding

An independent researcher will generate random numbers using an online tool (https://www.sealedenvelope.com/simple-randomiser/v1/lists) with a 1:1 allocation ratio, stratified by center. Randomization results will be stored in sealed opaque envelopes. Patients will be assigned to either the LP5 group or the placebo group. Postoperative care providers will prepare 5% lidocaine and placebo patches with patient numbers marked on the outer packaging. Patients, surgeons, anesthesia providers, outcome assessors, and statisticians will be blinded to group allocation.

### 2.8 Study interventions

In this study, patients will apply an LP5 or placebo patch for 12 h daily on the day before surgery and on postoperative days 1, 2, and 3. The preoperative patch will cover the anticipated incision site, while the postoperative patches will be applied 1.5 cm from either side of the incision. The placebo group will apply a placebo patch, identical in appearance and packaging to the actual medication patch. The lidocaine patches and placebo patches used in this study will be manufactured by Beijing Tide Pharmaceutical Co., Ltd. To maintain blinding, both patients and researchers will remain unaware of group assignments. The patches will be applied by medical professionals who are not involved in patient recruitment or data collection. Randomization will be performed before the study begins by an independent researcher using a computer-generated table of random numbers. Group assignments will be sealed in opaque envelopes to maintain blinding of the investigators, patients, and data analysts, and these envelopes will only be opened after the study is completed. The schedule of patient enrolment, study interventions, and outcome assessments will follow the SPIRIT statement (Table 1). All surgeries will be performed by experienced surgeons who have conducted at least 300 knee arthroplasty procedures prior to the study.

**TABLE 1:** Schedule of patient enrolment, study interventions and outcome assessment.

Time point	Study period								
	Enrolment	Allocation	Post-allocation					Close-out	Follow-up
	Pre-op visit	Pre-op 1 day	Intraoperatively	PACU	24 h post-op	48 h post-op	72 h post-op	Discharged	3 months
Patient enrolment									
Eligibility criteria	×								
Written informed consent	×								
Demographic data	×								
Baseline characteristics	×								
HADS	×								
PCS	×								
Randomization/allocation		×							
Study interventions									
5% Lidocaine Patch		×			×	×	×		
Placebo Patch		×			×	×	×		
Outcome assessment									
WOMAC-Pain Scale	×								×
WOMAC-Stiffness Scale	×								×
WOMAC-Function Scale	×								×
WOMAC-Total Scale	×								×
LANSS									×
SF-36									×
Post-op pain scores					×	×	×		
VAS Sleep score					×	×	×		
Post-op sufentanil use							×		
Need for rescue analgesia							×		
Length of PACU stay				×					
Length of hospital stay								×	
90-day mortality									×
Adverse Events									
Hypotension			×	×	×	×	×		
Hypertension			×	×	×	×	×		
Bradycardia			×	×	×	×	×		
Tachycardia			×	×	×	×	×		
Arrhythmia			×	×	×	×	×		
HD Events Intervention			×	×	×	×	×		
Headache		×			×	×	×		
Dizziness		×			×	×	×		
Nausea and vomiting		×		×	×	×	×		
Local skin allergy		×			×	×	×		
Wound infection					×	×	×		
Toxic reaction		×			×	×	×		

According to SPIRIT, statement of defining standard protocol items for clinical trials.

HADS, hospital anxiety and depression scale; PCS, pain catastrophizing scale; WOMAC, Western Ontario and McMaster Universities Osteoarthritis Index; LANSS, leeds assessment of neuropathic symptoms and signs; SF-36, Short Form Health Survey; Pre-op, Preoperative; Post-op, Postoperative; VAS, visual analogue scale; PACU, post-anaesthesia care unit; HD, haemodynamic; h, hours; SPIRIT, Standard Protocol Items: Recommendations for Interventional Trials.

### 2.9 Anaesthetic care

Intraoperative monitoring will include non-invasive blood pressure (NIBP), electrocardiography (ECG), pulse oximetry (SpO_2_), radial artery blood pressure, and nasopharyngeal temperature. Anesthesia will be induced with an intravenous injection of propofol at 1.5–2.5 mg/kg, combined with sufentanil at 0.2–0.4 μg/kg for analgesia. This will be followed by an intravenous injection of rocuronium at 0.6–0.9 mg/kg to achieve muscle relaxation. After induction, an endotracheal tube will be inserted, and its placement will be confirmed. The patients will receive mechanical ventilation, maintaining end-tidal carbon dioxide between 35 and 45 mmHg. Hypotension (mean arterial pressure <65 mmHg or a decrease of 20% from baseline) and bradycardia (heart rate <50 beats/min) will be treated as needed. A thermal blanket will be used during the operation to maintain nasopharyngeal temperature above 36°C. Additional doses of rocuronium will be administered as necessary to maintain muscle relaxation throughout the procedure. Continuous monitoring will include ECG, NIBP, SpO_2_, and bispectral index (BIS), with BIS values maintained between 40 and 60. Both groups of patients received 50 mg of flurbiprofen axetil during surgery. Postoperatively, all patients were transferred to the PACU. During their stay in the PACU and hospitalization, if a patient reported pain with an NRS score of 4 or higher, 5 μg of sufentanil was administered for analgesia, and the total dosage was recorded.

### 2.10 Data collection and monitoring

Data collection will be conducted byne characteristics, including preoperative medications, comorbidities, ASA score, smoking status, education level, and scores from the Hospital Anxiety and Depression Scale (HADS) and the Pain Catastrophizing Scale (PCS). All data will be documented in case report forms (CRFs) and subsequently entered into the electronic database under the supervision of the principal investigator. An independent Data Monitoring Committee (DMC) will continuously monitor the data collection process. Once data registration is complete, the electronic database will be secured. De-identified datasets will be sent to an independent statistician for final analysis following a predefined statistical plan. Any serious adverse events (SAEs), whether related or unrelated to the study medication (e.g., persistent hemodynamic instability), must be reported immediately to the principal investigator. In such cases, the perioperative care team should take the necessary measures to ensure the safety of the participants. These events must also be reported to the DMC within 24 h for further discussion and determination of whether modifications to the study interventions or termination of the study are necessary.

### 2.11 Sample size calculation

Based on previous studies, the standard deviation (SD) of the WOMAC Pain Scale prior to surgery is approximately 17, which corresponds to an 8–9 unit difference on the scale, representing the minimum clinically perceptible improvement (Wylde et al., 2015). To detect a 0.5 SD difference in WOMAC Pain Scale scores at 3 months postoperatively, with a two-sided α = 0.05 and 80% power, a sample size of 57 patients per group is necessary. Considering a potential dropout rate of 10%, we plan to enroll a total of 128 patients, with 64 patients in each group.

### 2.12 Statistical analysis

The normality of continuous variables will be assessed via the Shapiro-Wilk test. Normally distributed data will be presented as mean (standard deviation), whereas non-normally distributed data will be presented as median (interquartile range). For normally distributed continuous variables, independent t-tests or repeated measures ANOVA will be utilized for analysis. For non-normally distributed variables, the Mann-Whitney U test or generalized estimating equations (GEE) will be employed. Categorical data will be expressed as numbers (percentages) and analyzed via the chi-square test or Fisher’s exact test, depending on the expected frequencies. The primary outcome, the WOMAC-pain score at 3 months postoperatively, will be analyzed via a linear regression model, adjusting for baseline variables including HADS scores, PCS scores, baseline WOMAC-pain scores, sex, age, and BMI. Additionally, interactions between the treatment group and baseline scores of HADS, PCS, and WOMAC-pain will also be examined. Covariates with *p*-values <0.10 will be retained in the final model. Secondary outcomes will undergo multiple testing corrections via the Benjamini–Hochberg method, with the false discovery rate (FDR) significance level set at q < 0.05. Odds ratios (OR) and 95% confidence intervals (CI) will be reported where applicable. To ensure the robustness of our primary findings, we will conduct a sensitivity analysis using the bootstrap method. The bootstrap analysis will involve 1,000 resamples to estimate the regression coefficients and their 95% confidence intervals (CIs). This analysis will provide additional validation for the stability of the initial regression results. All results will be analyzed using a modified intention-to-treat approach, including all patients who undergo randomization and for whom relevant data are available. Statistical analyses will be conducted via SPSS software (version 25.0; IBM SPSS). A two-sided *p*-value <0.05 will be considered statistically significant unless specified otherwise for false discovery rate (FDR) corrections. Missing data will not be imputed, and no interim analyses are planned.

### 2.13 Patient and public involvement

Patients and the public will not participate in the study’s design, recruitment, conduct, or reporting. Study results will be shared with participants via email.

### 2.14 Principles and methods of unblinding or breaking the blind unblinding timeline

All participants will be unblinded following the completion of the study, specifically after all subjects have completed the 3-month follow-up period. Unblinding Method: The unblinding process will be overseen by an independent DMC. The DMC will oversee and retain all data related to randomization until the designated time for unblinding.

### 2.15 Emergency unblinding

In the event of an SAE or other emergency during the trial, unblinding will be performed immediately to facilitate appropriate medical intervention. In such cases, the principal investigator will contact the DMC for emergency unblinding, and the reasons for and process of unblinding will be thoroughly documented.

## 3 Discussion

This randomized, double-blind, placebo-controlled trial involves 128 adult patients across two centers and aims to evaluate the preventive effects of LP5 on CPSP following knee arthroplasty. The primary objective is to assess the efficacy of LP5 in reducing CPSP, as measured by the pain subscale of the WOMAC Index 3 months postoperatively. Secondary objectives include evaluating other aspects of postoperative pain and recovery, such as WOMAC stiffness, function, and total scales; the LANSS Pain Scale; the SF-36; postoperative pain scores; VAS sleep scores; postoperative sufentanil consumption; and the need for rescue analgesia. This study will be conducted in accordance with the Consolidated Standards of Reporting Trials (CONSORT) guidelines (Moher et al., 2012). To ensure the validity of the primary outcomes, baseline variables were adjusted, and sensitivity analyses were performed to assess the robustness of the findings. These methodological considerations aim to ensure the accuracy and reliability of the results.

LP5 has demonstrated significant efficacy in the management of both acute and chronic pain, and it is widely used in various conditions. In acute pain management, studies have shown that LP5 significantly reduces pain scores and the use of opioid analgesics following cesarean section (Queiroz et al., 2021). Additionally, LP5 has proven effective in managing pain after radical retropubic prostatectomy, laparoscopic ventral hernia repair, and robotic thoracic surgery (Habib et al., 2009; Saber et al., 2009; Vrooman et al., 2015). In chronic pain management, LP5 has shown exceptional efficacy in patients with postherpetic neuralgia (PHN) and diabetic neuropathy (DN). Patients with PHN using LP5 experienced significant reductions in pain intensity and affected areas, along with decreased demand for rescue medications (Mick and Correa-Illanes, 2012; Oscar et al., 2016). For DN patients, LP5 not only significantly improved pain intensity but also had a lower incidence of adverse events (Argoff et al., 2004; White et al., 2010). Furthermore, LP5 has been effective in managing chronic postoperative and post-traumatic neuropathic pain, reducing pain and affected areas, and improving patient function and quality of life (Hans et al., 2009; Hans et al., 2011). These findings highlight LP5’s broad applicability and significant impact in both acute and chronic pain management, suggesting its promising potential to improve postoperative outcomes and prevent the transition to chronic pain.

In knee arthroplasty, regional anesthesia techniques, including femoral and popliteal nerve blocks, are widely used for postoperative pain control. Although these techniques effectively alleviate postoperative pain, they also temporarily inhibit motor nerve function, potentially limiting early postoperative mobility and delaying functional recovery (Berninger et al., 2018; Wang et al., 2024). The inhibition of quadriceps function due to regional anesthesia increases the risk of falls and impedes rehabilitation (Hussain et al., 2023). Thus, none of the knee arthroplasty patients in this study received local anesthesia (LA), enabling an independent assessment of LP5’s effects. As a non-invasive, localized analgesic, LP5 offers effective pain control without impairing motor function, making it particularly advantageous within multimodal analgesia strategies. Its ease of application and targeted analgesic effects mitigate the risks associated with systemic analgesics or invasive techniques, offering considerable potential in postoperative pain management. LP5 may serve as a valuable adjunct to regional anesthesia or as an alternative for patients unsuitable for regional blocks, potentially accelerating recovery and improving overall outcomes.

Preclinical studies have demonstrated that the active ingredient in lidocaine patches, lidocaine, can effectively penetrate the stratum corneum to reach peripheral nerve endings (Gudin and Nalamachu, 2020). This process blocks persistent nociceptive signals, reduces neuronal sensitization, and decreases hyperexcitability in both the peripheral and central nervous systems (Smoker et al., 2019; Tsai et al., 2023). This mechanism is crucial in preventing the transition from acute to chronic pain. Specifically, lidocaine inhibits the activity of voltage-gated sodium channels, thereby reducing nociceptive input and preventing the transmission and amplification of pain signals. This theoretically aids in preventing the development of chronic pain (Vedantham and Cannon, 1999; Feizerfan and Sheh, 2014).

Given the high incidence and refractory nature of CPSP following knee arthroplasty (Beswick et al., 2012), this study aims to test the hypothesis that LP5 can prevent CPSP in patients undergoing knee arthroplasty. This rigorously designed study will evaluate the potential efficacy of lidocaine patches in managing postoperative pain, with a particular focus on their ability to prevent the transition from acute to chronic pain. Through this randomized controlled trial (RCT), we aim not only to provide critical data on the clinical application of lidocaine patches but also to offer new insights for improving future pain management strategies.

This study has several limitations. First, the choice of a 1.5 cm patch distance was based on safety considerations from previous studies and preliminary experiments. Although this distance is considered the safest for orthopedic surgeries, it may not be the optimal distance for analgesic efficacy. Nevertheless, safety is paramount in all clinical treatments. Second, the follow-up period of 3 months may not be sufficient to fully assess the long-term impact of CPSP. We plan to establish a specialized patient cohort for long-term follow-up and further research in this patient population. Additionally, the exclusion of certain patient groups, such as those with long-term opioid use and a history of alcoholism, may limit the generalizability and external validity of the findings. Including these patients in future research is important to provide a more comprehensive understanding of postoperative chronic pain, though their inclusion might introduce significant bias regarding CPSP outcomes.

The primary aim of this randomized controlled trial is to evaluate the effectiveness of the LP5 in preventing CPSP following knee arthroplasty. LP5 will be applied during the early perioperative period, including the day before surgery and postoperative days 1–3. The findings are expected to demonstrate that the use of LP5 may effectively prevent CPSP, reduce acute postoperative pain, and decrease opioid consumption. Additionally, LP5 may facilitate faster postoperative recovery, improving overall patient rehabilitation and quality of life. This study is expected to provide valuable insights for future multimodal analgesia strategies, optimizing postoperative pain management and improving patient outcomes.

## Data Availability

The original contributions presented in the study are included in the article/Supplementary Material, further inquiries can be directed to the corresponding authors.
